# Disrupted small-world brain network topology in pure conduct disorder

**DOI:** 10.18632/oncotarget.19098

**Published:** 2017-07-08

**Authors:** Feng-Mei Lu, Jian-Song Zhou, Jiang Zhang, Xiao-Ping Wang, Zhen Yuan

**Affiliations:** ^1^ Bioimaging Core, Faculty of Health Sciences, University of Macau, Macau SAR, China; ^2^ Mental Health Institute, Second Xiangya Hospital, Central South University, Hunan Province Technology Institute of Psychiatry, Key Laboratory of Psychiatry and Mental Health of Hunan Province, Changsha, China; ^3^ School of Electrical Engineering and Information, Sichuan University, Chengdu, China

**Keywords:** conduct disorder, resting-state functional magnetic resonance imaging, functional networks, small-world, graph theoretical analysis

## Abstract

**Objectives:**

Conduct disorder (CD) is characterized by the violation of the rights of others or basic social rules and a repetitive, persistent pattern of antisocial and aggressive behaviors. A large number of functional and structural neuroimaging studies have identified widely abnormalities in specific brain regions in CD, but the alterations in the topological organization of functional networks among them remain largely unknown.

**Methods:**

Resting-state functional magnetic resonance imaging was applied to investigate the intrinsic functional connectivity in 18 pure CD patients and eighteen typically developing healthy controls. We first constructed the functional networks and then examined the CD-related alteration in topology properties using graph theoretical analysis.

**Results:**

Both the CD group and healthy controls exhibited small-world topology. However, the CD group showed decreased global and local efficiency. Changes in the nodal characteristics in CD group were found predominantly in the default-mode network, visual, and striatum regions. In addition, altered fronto-limbic-striatum network topology was found to have a relationship with clinical scores.

**Conclusions:**

Our findings indicate the altered nodal topology of brain functional connectivity networks in CD.

**Significance:**

The results provide unequivocal evidence of a topological disruption in the brain networks that suggest some possible pathophysiological mechanisms underlying CD.

## INTRODUCTION

Conduct disorder (CD) is one of the most commonly diagnosed psychosocial disorders in childhood. The disorder is defined as a repetitive and persistent pattern of antisocial and aggressive behaviors in which the rights of others or basic social rules are violated (DSM-5, Diagnostic and Statistical Manual of Mental Disorders) [1]. CD presents a high risk for developing other mental health conditions (46% among male CD adolescents and 39% among females), such as substance abuse, major depression, attention-deficit/hyperactive disorder, antisocial personality disorder and suicide [2]. Previous community studies indicated a prevalence of 2-6% [3], and a meta-analysis of epidemiological studies showed a high prevalence of 3.2% for CD across countries in the age range of 6 to 18 years [4]. CD is also more prevalent in males compared with females [5]. Given the serious impact of CD and its prevalence, it is important to understand its underlying neural mechanisms.

Previous task-based and resting-state functional magnetic resonance imaging (rsfMRI) studies have shown that the neural mechanisms of CD are associated with functional impairments in widespread brain regions, including the insula [6–8], anterior cingulate cortex [8], amygdala [6, 9], fusiform gyrus [7], and orbitofrontal cortex [10]. Moreover, structural neuroimaging studies found that compared with healthy controls, those with CD had reduced gray matter volume in the temporal lobes, orbitofrontal regions, amygdala, and other limbic and paralimbic regions [11, 12]. These altered regions are also involved in various brain functional networks, including the default-mode network (DMN), the somatosensory network, and the visual network [13–15]. There is accumulating evidence demonstrating the abnormalities in widespread regions and networks in CD, but few studies have investigated the small-world topological properties of functional networks of the whole brain in CD.

Graph theoretical analysis is an alternative data-driven approach that has recently allowed us to quantify the topological properties of disconnectivity in brain networks independently of *a priori* seeds. The method models the brain as a large-scale network that is represented graphically by a series of nodes (brain regions) and edges (functional connections between pairs of nodes) [16]. Thus, the various graph theoretical properties can be used to investigate the inter-connectedness of the whole network, as well as how a single region interacts with the remaining regions of brain. More importantly, graph theoretical analysis has provided novel insights to explore functional abnormalities in CD at both the global and nodal levels inside or outside classical CD-related regions. Using this approach, Watts and Strogatz demonstrated that graphs with higher clustering and similar shortest path length compared with a random network can be characterized as small-world networks [17]. Clustering is the ratio of the number of existing interconnections of a node with its neighbors to the maximum of all possible connections, while the path length is the average number of minimum links that are required to travel between any two nodes. At the nodal level, the node degree measures the connectedness of an isolated node with all the other nodes, which can identify highly connected nodes that may play important roles in information integration in a network.

Recently, disrupted small-world properties have been found in numerous brain diseases, such as depression [18, 19], schizophrenia [20], Alzheimer's disease [21], epilepsy [22], obsessive-compulsive disorder (OCD) [23], autism [24], attention-deficit/hyperactivity disorder (ADHD) [25], multiple sclerosis [26], post-traumatic stress disorder [27], and spinal cord injury [28]. We hypothesized that CD patients would show disrupted functional topological organization. To test this, we applied rsfMRI to construct the brain functional connectomes in 18 adolescents with pure CD and 18 precisely age-, and gender-matched typically developing (TD) healthy controls. The rsfMRI was used to explore the intrinsic brain activity based on the spontaneous low-frequency (0.01-0.1 Hz) fluctuations in the signal depending on the blood oxygen level during a resting state [29–32]. The graph theoretical approach was then used to investigate the topological organization of the functional connectivity networks. The individual functional connectivity matrixes were reconstructed by computing the correlations between the mean time series of two brain regions. Finally, the CD group was compared with the TD group with respect to the global and nodal network properties, and the clinical relevance of the aberrant brain network topologies were evaluated.

The recruited participants were only pure CD patients without any comorbid psychiatric disorders, such as substance use disorder (SUD), anxiety, depression, OCD, oppositional defiant disorder (ODD), ADHD, affective disorders, alcohol- and drug-use disorder, or mental retardation. This choice was largely due to the significant differences between pure CD patients and healthy subjects [8, 33–35]. In addition, only boys with pure CD were included because previous investigations on neurological and psychiatric disorders showed substantial differences between male and female brains [36, 37].

## RESULTS

### Demographic and clinical comparisons between groups

Table 1 provides information on the demographics and clinical variables from the two groups. The total score, attention score, and non-planning score of the Barratt Impulsivity Scale (BIS) were significantly different between the CD and TD groups (*p* < 0.05; Table 1).

**Table 1:** Demographic features and clinical data of CD patients and TD subjects

	CD (*n* = 18)	TD (*n* = 18)	*t-*value	*df*	*p-*value
Age (yrs)	16.1 ± 0.54	15.9 ± 0.32	1.124	34	0.27
Education (yrs)	9.4 ± 2.0	9.2 ± 1.9	0.7	34	0.47
Mother’s education (yrs)	8.2 ± 4.1	10.1 ± 3.5	-1.6	34	0.13
Father’s education (yrs)	8.8 ± 2.6	10.4 ± 2.2	-1.9	34	0.07
BIS-11					
Total score	76.06 ± 8.26	64.89 ± 11.14	3.417	34	< 0.001
Attention subscale score	18.39 ± 2.06	17.83 ± 3.67	0.560	34	0.58
Motor subscale score	26.89 ± 4.76	20.61 ± 4.16	4.211	34	< 0.001
Non-planning subscale score	30.78 ± 4.43	26.44 ± 5.24	2.681	34	0.01

Values are presented as mean ± SD. The *p* value was obtained by two-sample *t*-tests. CD, conduct disorder; TD, typically developing; SD, standard deviation; BIS-11, Barratt Impulsivity Scale-11; yrs, years.

### Global topological organization of the brain functional networks

**Figure 1:**
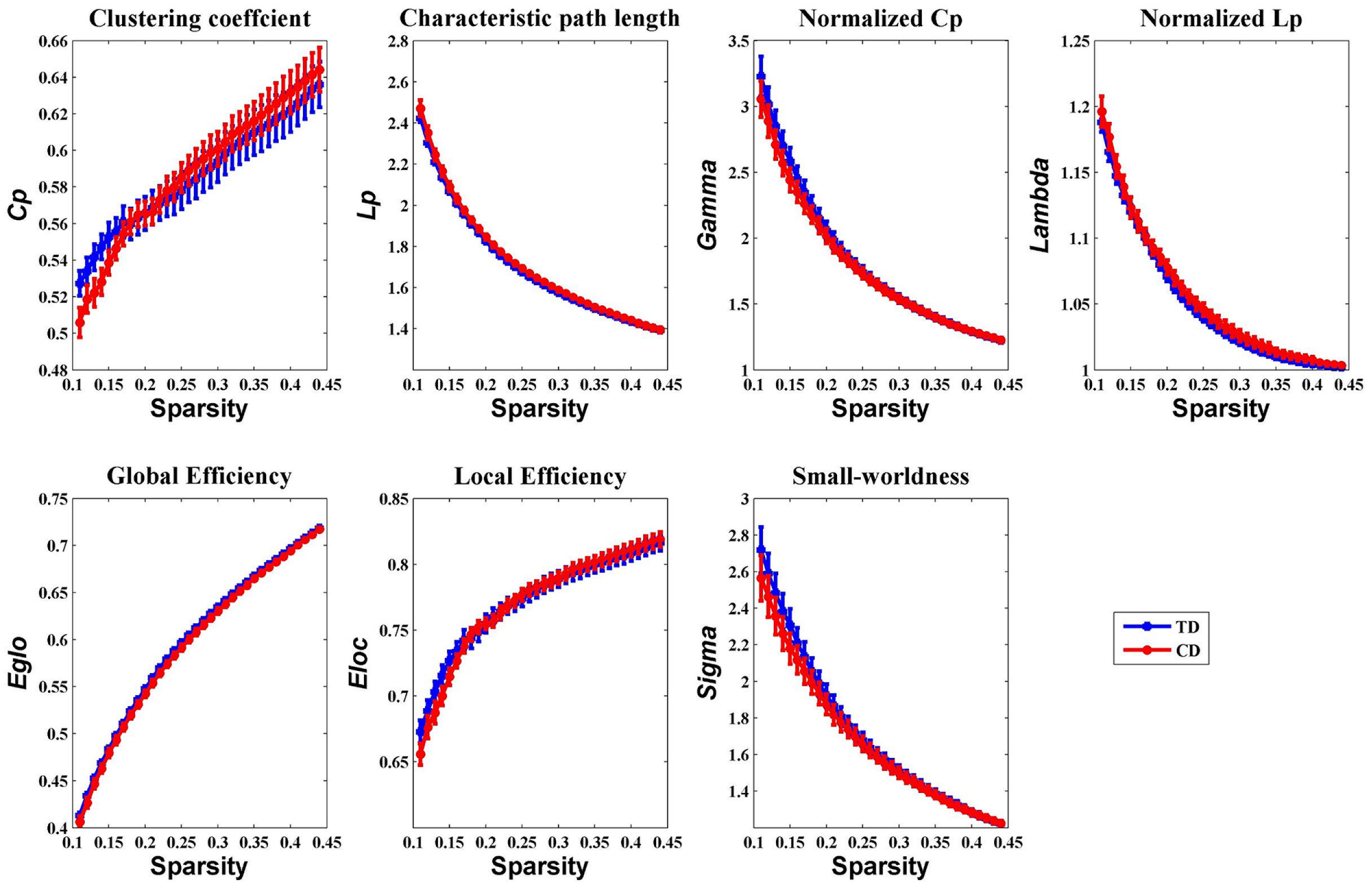
Group comparison of global network topological properties (**C**_*p*_**, *L***_*p*_**, *E***_*glo*_**, *E***_*loc*_, λ, γ, and σ) between the CD and TD group (two-sample *t*-tests, *p* < 0.05, uncorrected) The small-worldness suggests a small-world topology for functional brain networks of both the CD and TD group. The error bar represents the standard error (SE) of the mean. There were no significant differences in the global network properties between the two groups. Cp, clustering coefficient; Lp, path length; Eglo, global efficiency; Eloc, local efficiency; CD, conduct disorder; TD, typically developing.

**Figure 2:**
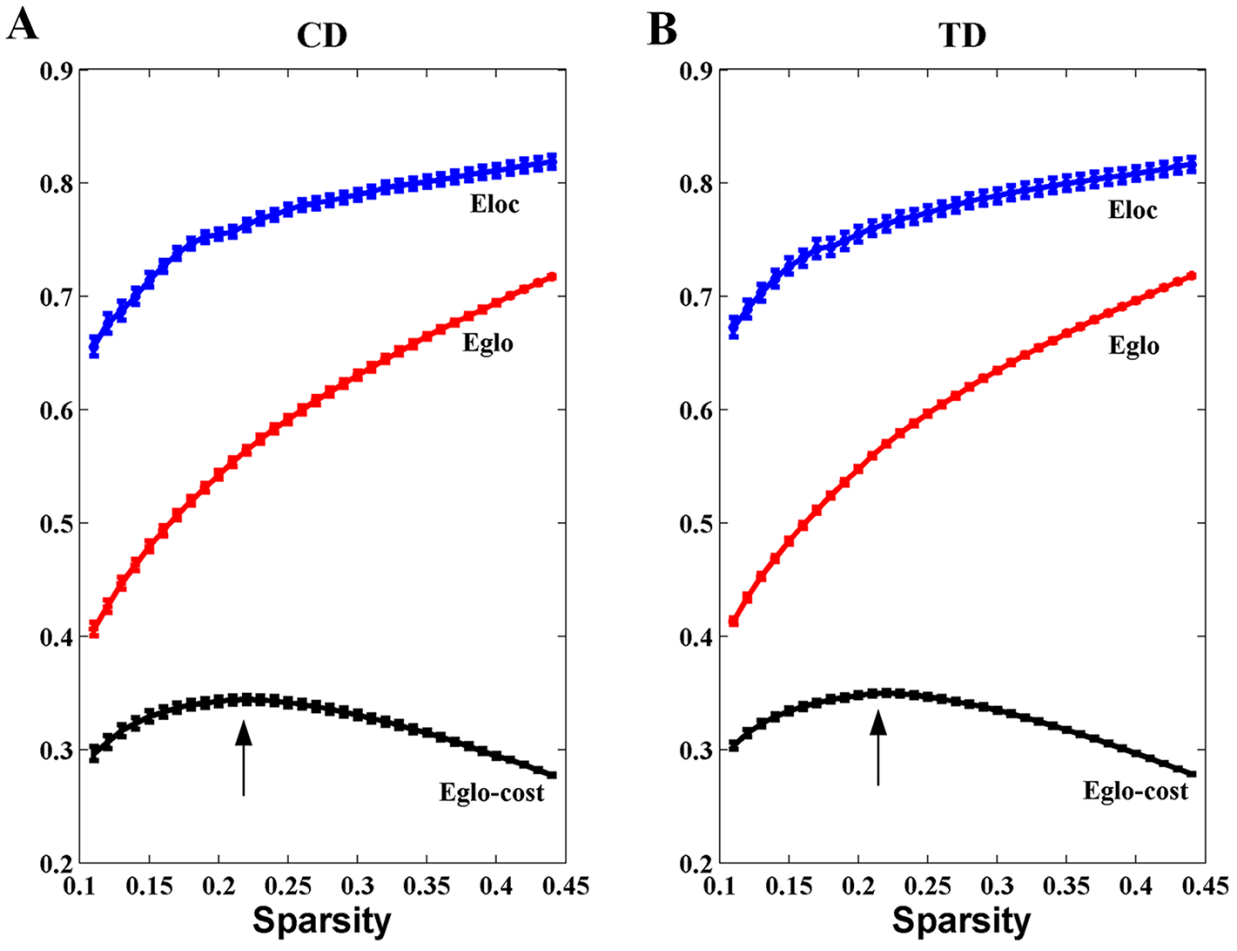
Economical properties of brain functional networks The global and local efficiency of brain networks in both CD and TD groups increase monotonically with the sparsity (cost). The cost efficiency (the difference between the global efficiency and cost, i.e., Eglo-cost) has a maximum value at the cost of about 0.20 (the black arrows). The error bar represents the standard error (SE) of the mean.

**Figure 3:**
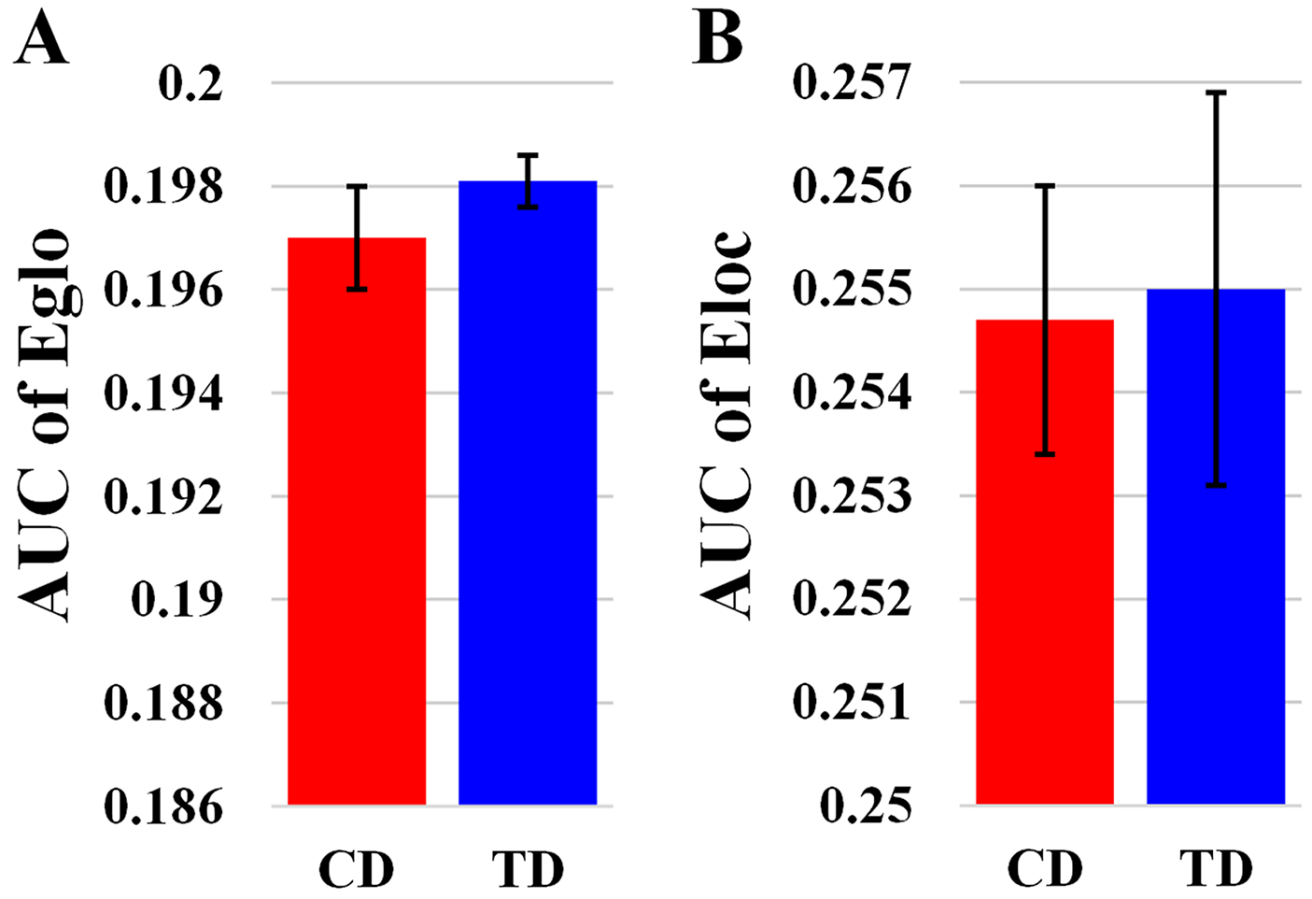
Bar-plot showing the mean and SE for the AUC of global efficiency **(A)** and the AUC of local efficiency **(B)** in both CD and TD group. Eglo, the global efficiency; Eloc, the local efficiency. The error bar represents the standard error (SE) of the mean.

Overall, the sigma over the threshold ranged from 0.11 to 0.44 in both the CD and TD groups was greater than 1.1. This demonstrates that both groups had small-world topology in the brain functional networks (Figure 1). However, there were no significant differences in all the global network properties through all the sparsity between the two groups. Additionally, Figure 2 shows that the cost efficiency (i.e., $E_{glo}-\text{cost}$) of brain networks in both groups was above zero across all sparsity thresholds and reached a maximum value at sparsity of about 0.20, as indicated by black arrows, which showed the economical properties of brain networks. Our results are in accordance with those by Achard et al. [38]. Furthermore, the brain networks of the CD group demonstrated decreased global efficiency and local efficiency in comparison with the TD group (Figure 3).

### Altered regional topological organization of brain functional networks in CD

**Figure 4:**
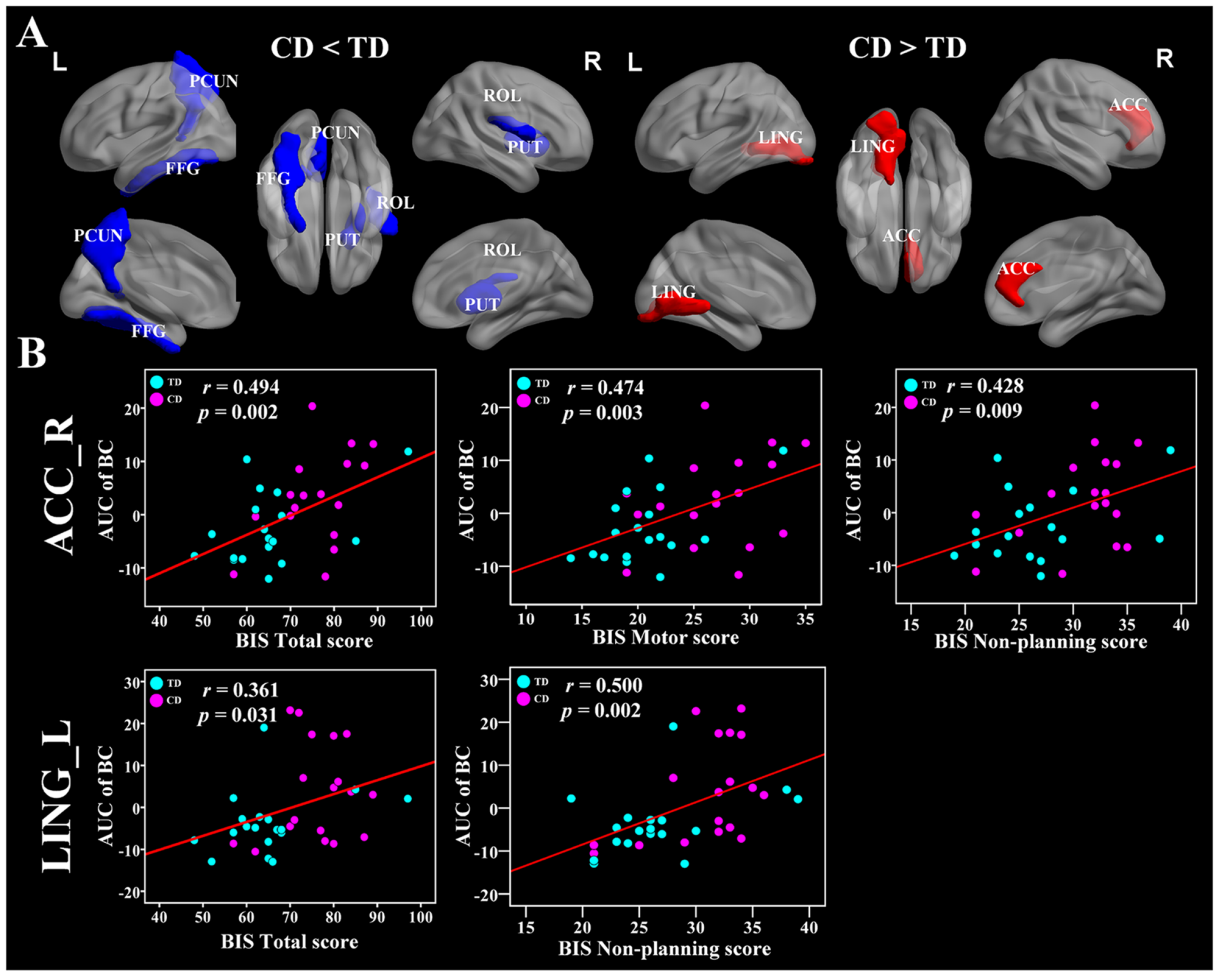
Brain regions showing disrupted nodal network properties in brain functional networks in CD group and their relationships with clinical variables **(A)** Regions with abnormal nodal network properties in CD patients as compared with TD group. Group comparisons were based on permutation tests (5,000 permutations, *p* < 0.05, controlling for the age, and mean FD). The blue and red regions represent significantly decreased and increased nodal network properties in CD group as compared with TD group, respectively. Results were visualized with the BrainNet Viewer (http://www.nitrc.org/projects/bnv/). See Table 2 for the detailed information. **(B)** Significant relationships between the AUC of nodal network properties and BIS-11 scores, controlling for the age, and mean FD (*p* < 0.05). The red color means positive correlations. The magenta and cyan circles represent the BIS scores of CD and TD, respectively. CD, conduct disorder; TD, typically developing; AUC, area under the curve; PCUN, precuneus; FFG, fusiform gyrus; PUT, putamen; ROL, rolandic operculum; ACC, anterior cingulate cortex; LING, lingual gyrus; BIS, Barratt Impulsivity Scale; L, left hemisphere; R, right hemisphere.

We identified several brain regions showing significant between-group differences in the CD group in the nodal betweenness centrality, nodal degree, and nodal efficiency (*p* < 0.05, uncorrected) (Figure 4A and Table 2). Compared with the TD group, the CD group showed decreased nodal betweenness centrality in the right rolandic operculum (ROL), left fusiform gyrus (FFG), and left precuneus (PCUN), as well as decreased nodal efficiency in the right ROL (Figure 4A and Table 2). Increased nodal betweenness centralities were found in the left lingual gyrus (LING) and the right anterior cingulate cortex (ACC) in the CD group compared with the TD group (Figure 4A and Table 2). Importantly, the left LING and right ACC were positively correlated with BIS-11 scores in the regional nodal betweenness centrality (Figure 4B).

**Table 2:** Nodal network topology in CD group compared with TD group

Brain region	Node topology	CD	TD	CD *vs.* TD	*p-*value
R ROL	AUC of BC	9.59 ± 5.74	13.42 ± 5.26	↓	0.040
L FFG	AUC of BC	13.49 ± 9.26	21.69 ± 13.29	↓	0.046
L PCUN	AUC of BC	11.96 ± 6.89	16.78 ± 9.43	↓	0.044
R ROL	AUC of E_nodal_	0.19 ± 0.02	0.20 ± 0.01	↓	0.012
L LING	AUC of BC	17.25 ± 11.73	10.20 ± 7.53	↑	0.026
R ACC	AUC of BC	16.50 ± 8.97	11.50 ± 7.10	↑	0.046

Group comparisons were based on permutation tests controlled for age and mean FD, *p* < 0.05, 5000 permutations. The AUC of the nodal topology was calculated over the range of 0.11 ≤ T ≤ 0.44 with an interval of 0.01. The AUC of the nodal topology scores are reported as mean ± SD. CD, conduct disorder; TD, typically developing; L, left hemisphere; R, right hemisphere; ROL, rolandic operculum; FFG, fusiform gyrus; PCUN, precuneus; LING, lingual gyrus; ACC, anterior cingulate cortex; AUC, area under the curve; BC, nodal betweenness centrality; E_nodal_, nodal efficiency; ↓, decreased; ↑, increased.

### Relationships between the aberrant nodal network topology and BIS-11 scores

**Figure 5:**
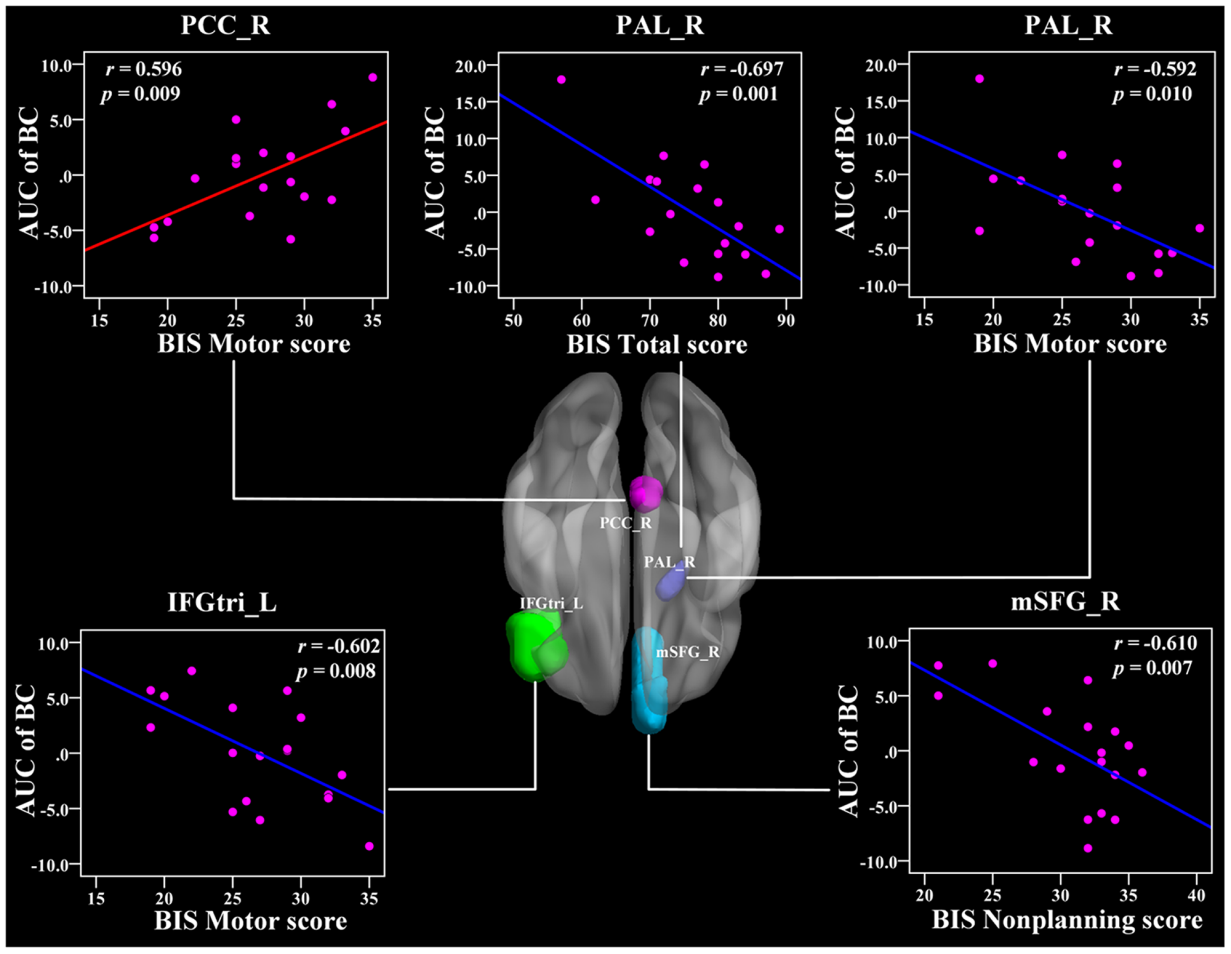
The Pearson correlation between the AUC of the nodal betweenness centrality with BIS-11 scores in CD group (*p* < 1/90, uncorrected, controlling for the age, and mean FD) The AUC of each nodal topology was calculated over the range of 0.11 ≤ T ≤ 0.44 with an interval of 0.01. The red color reveals the positive correlation while the blue color represents the negative correlation. For more detailed information see Table 3. BIS, Barratt Impulsivity Scale; L, left hemisphere; R, right hemisphere; AUC, area under the curve; BC, nodal betweenness centrality; CD, conduct disorder; PCC, posterior cingulate cortex; PAL, pallidum; IFGtri, inferior frontal gyrus pars triangularis; mSFG, medial superior frontal gyrus.

No significant correlations were found between the global network metrics (*C_p_, L_p_, E_glo_, E_loc_*, *λ*, *γ*, and *σ*) and the BIS-11 scores. In the area under the curve (AUC) of nodal betweenness centrality, the right posterior cingulate cortex (PCC) was positively related with the BIS motor score (r = 0.596, *p* < 0.011) (Figure 5 and Table 3). The left ACC and the right cuneus (CUN) were also positively associated with BIS-11 scores (*p* < 0.05, uncorrected) (Table 3). The right pallidum (PAL), left inferior frontal gyrus pars triangularis (IFGtri), and medial superior frontal gyrus (mSFG) were negatively correlated with BIS-11 scores (*p* < 0.011) (Figure 5 and Table 3). The left middle temporal gyrus (MTG), left PAL, and right supramariginal gyrus (SMG) were also negatively linked with BIS-11 scores (*p* < 0.05, uncorrected) (Table 3).

**Table 3:** Significant correlations between nodal network topology and the BIS-11 scores in CD group

Relationships	Region of interest	BIS-11 score subtype	r*-*value	*p*-value
***Relationships between AUC of BC and BIS-11 scores***				
**Positive**	L ACC	BIS Total Score	0.507	0.032
	R PCC	BIS Total Score	0.538	0.021
	R CUN	BIS Attention Score	0.542	0.020
	R PCC	BIS Motor Score	0.596	0.009^*^
	L ACC	BIS Non-planning Score	0.500	0.035
**Negative**	R PAL	BIS Total Score	-0.697	0.001^*^
	L MTG	BIS Attention Score	-0.488	0.040
	L IFGtri	BIS Motor Score	-0.602	0.008^*^
	L PAL	BIS Motor Score	-0.506	0.032
	R PAL	BIS Motor Score	-0.592	0.010^*^
	R mSFG	BIS Non-planning Score	-0.610	0.007^*^
	R SMG	BIS Non-planning Score	-0.551	0.018
	R PAL	BIS Non-planning Score	-0.493	0.037
***Relationships between AUC of Deg and BIS-11 scores***				
**Positive**	R PCC	BIS Total Score	0.490	0.039
	L PUT	BIS Attention Score	0.477	0.045
	R PUT	BIS Attention Score	0.589	0.010^*^
	R PCC	BIS Motor Score	0.633	0.005^*^
	R ANG	BIS Motor Score	0.587	0.010^*^
	L ACC	BIS Non-planning Score	0.483	0.042
	L PAL	BIS Non-planning Score	0.488	0.040
**Negative**	L PreCG	BIS Total Score	-0.511	0.030
	L IFGtri	BIS Total Score	-0.627	0.005^*^
	L IFGorb	BIS Total Score	-0.522	0.026
	L AMYG	BIS Total Score	-0.591	0.010^*^
	R mSFG	BIS Attention Score	-0.504	0.033
	R MTG	BIS Attention Score	-0.611	0.007^*^
	L IFGtri	BIS Motor Score	-0.592	0.010^*^
	L IFGorb	BIS Motor Score	-0.498	0.035
	L AMYG	BIS Motor Score	-0.667	0.002^*^
	L STG	BIS Motor Score	-0.563	0.015
	R SMG	BIS Non-planning Score	-0.469	0.049
***Relationships between AUC of E***_***nodal***_ ***and BIS-11 scores***				
**Positive**	R PCC	BIS Total Score	0.521	0.027
	L CAL	BIS Total Score	0.480	0.044
	R PUT	BIS Attention Score	0.541	0.020
	R PCC	BIS Motor Score	0.636	0.005^*^
	R ANG	BIS Motor Score	0.645	0.004^*^
	L ACC	BIS Non-planning Score	0.490	0.039
**Negative**	L PreCG	BIS Total Score	-0.479	0.044
	L IFGtri	BIS Total Score	-0.544	0.020
	L IFGorb	BIS Total Score	-0.505	0.033
	L AMYG	BIS Total Score	-0.521	0.027
	R mSFG	BIS Attention Score	-0.515	0.029
	R MTG	BIS Attention Score	-0.626	0.005^*^
	L IFGtri	BIS Motor Score	-0.543	0.020
	L IFGorb	BIS Motor Score	-0.489	0.039
	L AMYG	BIS Motor Score	-0.623	0.006^*^
	L STG	BIS Motor Score	-0.578	0.012

Pearson correlation analyses were corrected controlling for the age, and mean FD. The AUC of the nodal topology was calculated over the range of 0.11 ≤ T ≤ 0.44 with an interval of 0.01. L, left hemisphere; R, right hemisphere; AUC, area under the curve; BC, nodal betweenness centrality; Deg, nodal degree; E_nodal_, nodal efficiency; BIS, Barratt Impulsivity Scale; CD, conduct disorder; TD, typically developing; ACC, anterior cingulate cortex; PCC, posterior cingulate cortex; PAL, pallidum; MTG, middle temporal gyrus; IFGtri, inferior frontal gyrus pars triangularis; mSFG, medial superior frontal gyrus; SMG, supramarginal gyrus; PUT, putamen; ANG, angular gyrus; PreCG, precentral gyrus; IFGorb, inferior frontal gyrus pars orbitalis; AMYG, amygdala; mSFG, medial superior frontal gyrus; STG, superior temporal gyrus; AUC, area under the curve; *Reported results are significant for *p* < 1/90 based on false positive correlation for multiple comparisons.

**Figure 6:**
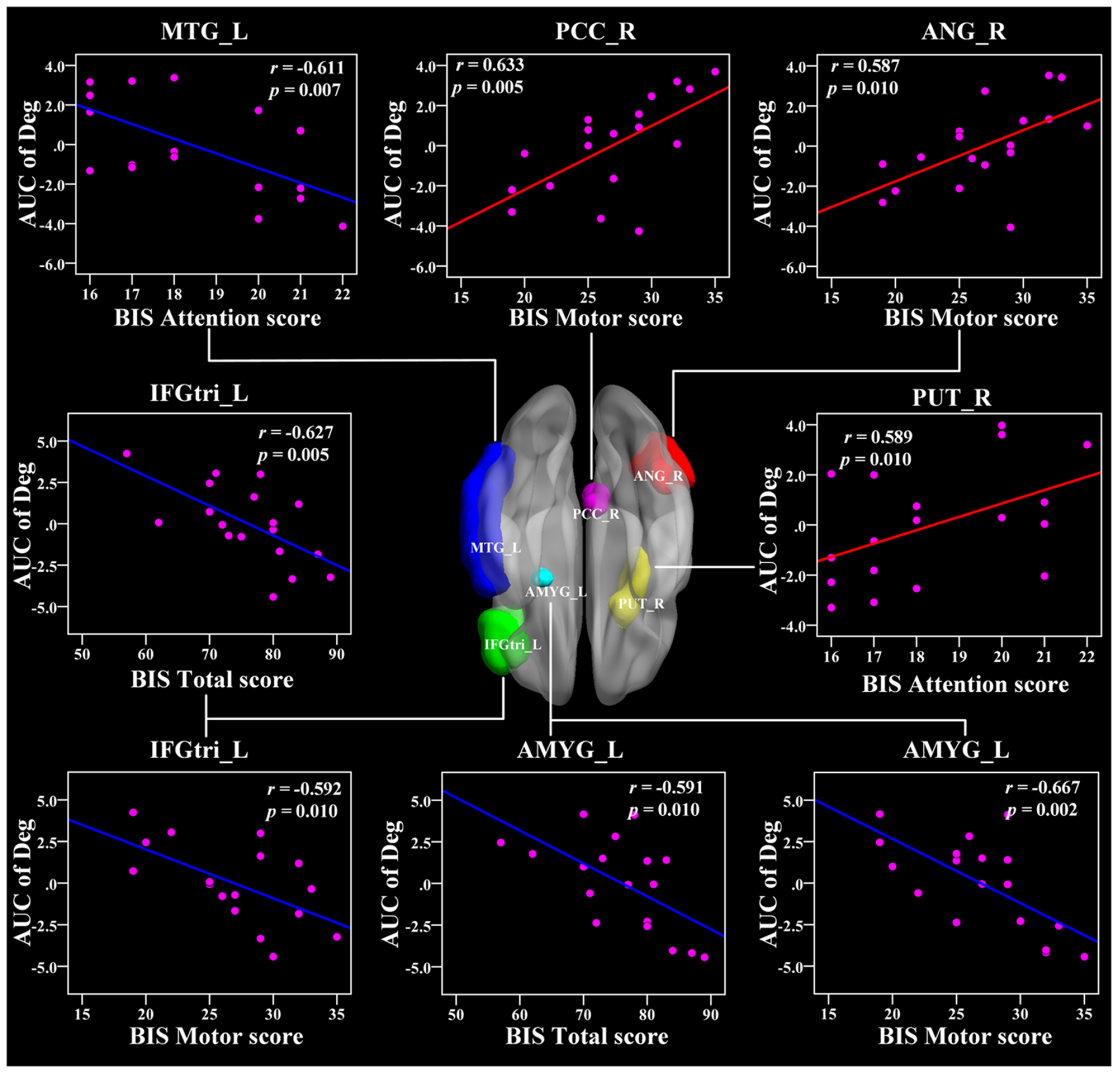
The Pearson correlation between the AUC of the nodal degree with BIS-11 scores in CD group (*p* < 0.011, uncorrected, controlling for the age, and mean FD) The AUC of each nodal topology was calculated over the range of 0.11 ≤ T ≤ 0.44 with an interval of 0.01. The red color reveals the positive correlation while the blue color represents the negative correlation. For more detailed information see Table 3. BIS, Barratt Impulsivity Scale; L, left hemisphere; R, right hemisphere; AUC, area under the curve; Deg, nodal degree; CD, conduct disorder. MTG, middle temporal gyrus; PCC, posterior cingulate cortex; ANG, angular gyrus; IFGtri, inferior frontal gyrus pars triangularis; PUT, putamen; AMYG, amygdala.

In the AUC of nodal degree, the right PUT, right PCC, and right angular (ANG) were positively related with BIS-11 scores (r = 0.589, 0.633, 0.587, respectively, *p* < 0.011) (Figure 6 and Table 3). In addition, the left PUT, left ACC, and left PAL were also positively associated with BIS-11 scores (Table 3). The left IFGtri, left amygdala (AMYG), and right MTG were related with BIS-11 scores (*p* < 0.011) (Figure 6 and Table 3). The left precentral gyrus (PreCG), right mSFG, left inferior frontal gyrus pars orbitalis (IFGorb), left superior temporal gyrus (STG), and right SMG were also negatively related with BIS-11 scores (*p* < 0.05, uncorrected) (Table 3).

**Figure 7:**
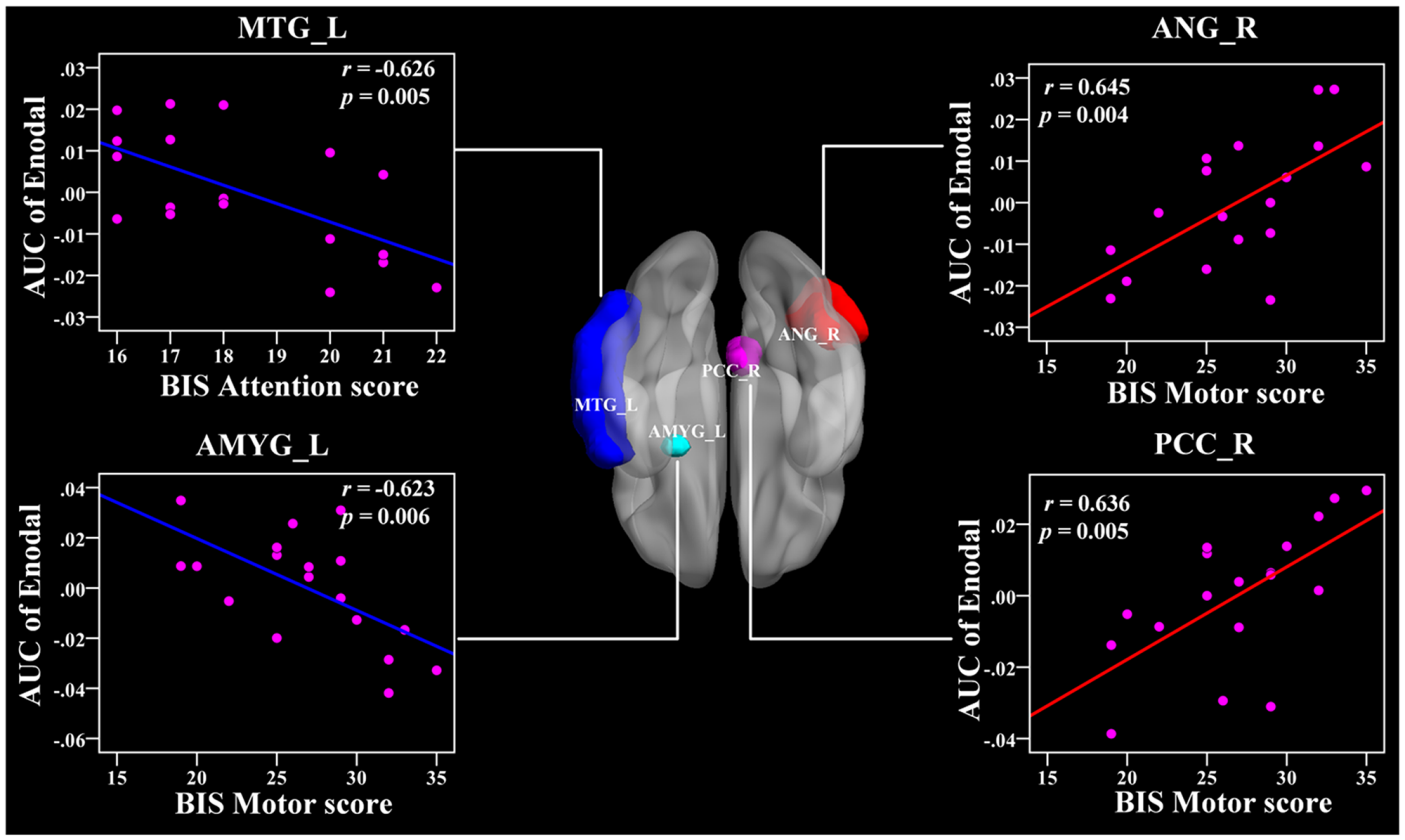
The Pearson correlation between the AUC of the nodal efficiency with BIS-11 scores in CD group (*p* < 0.011, uncorrected, controlling for the age, and mean FD) The AUC of each nodal topology was calculated over the range of 0.11 ≤ T ≤ 0.44 with an interval of 0.01. The red color reveals the positive correlation while the blue color represents the negative correlation. For more detailed information see Table 3. BIS, Barratt Impulsivity Scale; L, left hemisphere; R, right hemisphere; AUC, area under the curve; E_nodal_, nodal efficiency; CD, conduct disorder. MTG, middle temporal gyrus; ANG, angular gyrus; AMYG, amygdala; PCC, posterior cingulate gyrus.

In the AUC of nodal efficiency, the right PCC and right ANG were positively related with BIS-11 scores (r = 0.636, 0.645, respectively, *p* < 0.011) (Figure 7 and Table 3). The left CAL, right PUT, and left ACC were also positively correlated with BIS-11 scores (Table 3). The left AMYG and right MTG were negatively associated with BIS-11 scores (*p* < 0.011) (Figure 7 and Table 3). The left PreCG, left IFGtri, left IFGorb, right mSFG, and left STG were also negatively linked with BIS-11 scores (*p* < 0.05, uncorrected) (Table 3).

## DISCUSSION

To our knowledge, this is the first study to investigate the small-world properties of the brain functional networks in adolescents with pure CD. At the global level, our results showed that both the CD group and the healthy controls exhibited small-world topology in the brain functional networks. However, those in the CD group showed slightly lower global and local efficiency than healthy subjects, suggesting a disruption of the brain network and a shift towards regular networks. At the nodal level, our results revealed that the nodal characteristics in the CD group were significantly altered in several regions compared with healthy controls, mainly in the default-mode regions, visual-related regions, and the striatum. Furthermore, the default-mode regions, prefrontal cortex, temporal cortex, emotion-related regions, and striatum were linked with the BIS-11 scores. Our results provided unequivocal evidence of topological alterations in the brain networks, suggesting some possible pathophysiological mechanisms underlying CD.

### Small-world topology in CD adolescents

Both the CD and the TD groups displayed small-world architecture properties in the functional networks (Figure 1). The small-world network induced by Watts and Strogatz included the features of high clustering and short path length [17]. Both groups exhibiting the small-world topology in the present study provides further evidence that this type of brain architecture is robust to developmental aberration or disease [39]. Furthermore, the functional networks also revealed economical properties (Figure 2) in both the CD and TD groups. This suggests that the brain network tends to process information efficiently at a low cost, which is in agreement with a previous study [38].

Although both groups showed small-world topology, the global and local efficiency was reduced in the CD group, suggesting a disruption of the brain network and a shift towards regular networks. Previous evidence suggests that the speed of information propagation and the synchronizability across distant regions is weaker in regular lattices than in economical small-world networks [40]. Thus, the abnormalities in the small-world network caused by brain disorders may reflect a less optimal network topology in patients with CD. No significant changes were found in the global network properties, suggesting that CD is related to the disruptive integrity of the specific brain regions rather than the whole-brain network. Taken together, our findings provide support that the CD-related network shift may cause alterations to the network topology.

### CD-related alterations in nodal topology

The nodal degree measures the extent to which a node is connected to the rest of the other nodes in a network, while the nodal efficiency evaluates the level of information propagation of a node with all other nodes in the network. The nodal betweenness estimates the influence of a node over information flow with the rest of the nodes in a network [38]. Using these measurements, we identified abnormal nodal characteristics in several brain regions in the CD group (Table 2), which indicated alterations in the activation of the regions.In particular, the CD group exhibited decreased nodal betweenness centrality compared with healthy controls in the right ROL, left FFG, and left PCUN, as well as decreased nodal efficiency in the right ROL. Increased nodal betweenness centrality occurred in the left LING and the right ACC in the CD group.

The PCUN is one of the key regions of the DMN, a network that is important for self-referential mental thought cognition when a person is free from a task [41]. The decreased nodal betweenness centrality in the PCUN was in accordance with previous task-based and resting-state fMRI studies that suggest reduced activity in this region in CD patients [14, 33, 35, 42]. Our findings of decreased nodal betweenness centrality in the PCUN suggest that the hypo-activity in the PCUN serves as a pathological mechanism of CD from a new perspective of information flow.

The FFG and LING are critical for the visual systems, and dysfunctions can contribute to dysfunctions in low-order cognitive processing in CD [15, 43]. Importantly, the FFG plays an essential role in facial identity processing and facial expression perception [44]. Previous structural studies including 63 CD subjects demonstrated that CD is associated with reduced gray matter volume in the FFG compared to the healthy controls [12, 45]. The ACC is vital in emotional monitoring processes and cognitive processes such as regulation of decision making and conflict response [46, 47], and dysfunctions in this region were observed in CD [48]. One could speculate that the altered nodal betweenness centrality in this region might reflect an impaired capability of emotional behavior control effort in patients with CD and may thus result in an increased tendency for impulsive aggression. Our results of CD-related regional changes in the nodal characteristics suggested that the changes in nodal roles of the visual networks and profoundly affected by this disorder.

### Aberrant nodal topology in the fronto-limbic-striatum network is related to impulsivity

We also found relationships between the BIS-11 scores and the nodal topology in the limbic system (PCC, AMYG), striatum (PUT), and frontal regions (IFGtri) in CD patients. The PCC is the pivotal node of the DMN and is densely structurally connected with widespread brain regions [49, 50]. It serves various roles, including the processing of the salience of events and faces, memory recollection, self-relevance assessment, and a more active role in the regulation of cognition, both at rest and during cognitively challenging tasks [51–54]. Our findings showed a positive relationship between the PCC and the BIS-11 score, suggesting that the disruption of the information integration ability of the DMN is closely associated with impulsivity in CD patients, which may serve as a neural mechanism underlying CD.

In addition, we found a negative association between the nodal betweenness and nodal degree in the IFG and BIS-11 score, indicating that higher impulsivity is associated with a weaker IFG hubness for information integration. Previous evidence has shown that antisocial behavior in children might be due to deficiencies in the brain's self-control functions, which are called the executive functions [55]. The frontal cortex and its connective pathways with other brain regions play critical roles in executive function [56]. In particular, the left IFG is mainly engaged in inhibitory control processes [57]. Thus, the altered nodal topology in the IFG in this study may serve as a neural mechanism for impulsivity in CD.

Importantly, the putamen is a key component of the fronto-striatum circuits. Concerning the IFG and putamen, we concluded that the topology of the fronto-striatum connectivity developed distinctively in CD, which suggests that the fronto-striatum network abnormality contributed to the neural circuitry pathophysiology underlying CD. Furthermore, the CD is predominantly characterized by abnormalities in the lateral orbital and ventromedial prefrontal cortices, superior temporal lobes, and underlying limbic structures including the ACC, AMYG, insula, hippocampus, hypothalamus, ventral striatum, and other connected areas that mediate hot cognitive functions regulating motivation and emotion [11].

The AMYG is involved in negative affect and threat processing, and together with the ventral striatum, it mediates stimulus-reward associations and motivation functions [58, 59]. Recently, using structural neuroimaging with 23 CD patients, Huebner et al. demonstrated that CD patients had reduced total gray matter volumes (GMVs) mainly in the bilateral temporal lobes, limbic regions (the left AMYG and hippocampus), and frontal regions compared with healthy controls. The hyperactivity/impulsiveness symptoms were also negatively related with GMVs in the left IFG [12]. Another structural study performed by Sterzer et al. showed that patients with CD exhibited reduced GMVs in the bilateral insula and left AMYG relative to healthy subjects, and both of them were associated with aggressive and inattentive symptoms [60]. More recently, an fMRI study found that CD was associated with a reduced activation to fearful faces in the AMYG as compared with healthy subjects [61]. Moreover, lesion, animal, and imaging studies have suggested that the orbitofrontal and temporal lobes are important for impulsivity and aggression [62, 63]. Our results may suggest abnormalities in the executive functions and aberrant function connectivity in the fronto-limbic-striatum systems of CD patients.

To evaluate the current results appropriately, some issues should be addressed. First of all, we constructed the functional brain networks using a prior template based on the AAL atlas to parcellate the whole brain into 90 regions. Previous studies suggest that different parcellation strategies may affect our results. In future studies, other appropriate parcellation schemes could be adopted to map the brain network topology in CD. Second, a relatively weak correction strategy was used in this study because of our relatively small sample size of CD adolescents. For this reason, further studies need to be performed using a larger sample of CD patients to confirm our exploratory findings. Finally, we caution against overgeneralization of our results. The current study was restricted to male adolescents, so our results might not generalize to female adolescents. Additionally, we studied only CD patients with non-comorbid diagnoses (such as ADHD and ODD), which limited the ability to extend our findings to those with other comorbid conditions.

## MATERIALS AND METHODS

### Participants

The participants were 18 right-handed adolescents with pure CD (mean age 16.1 ± 0.5 years, age range 15-17 years) and 18 precisely age-, handedness-, and gender-matched healthy controls, as described in detail elsewhere [7, 14, 15, 64]. The CD adolescents were recruited from the Hunan Province Youth Detection Centre, while the TD controls were recruited from schools in the local community. The CD patients in our study were childhood-onset CD who were younger than 10 years old at the time of symptom onset [1]. Oral and written information about the aims, content, duration of interviews, and scanning procedures were told to all the CD and TD individuals and their parents or legal guardians. Written informed content for study participation and parental permission was obtained from all the participants as well as their parents or legal guardians prior to our research. The present study was approved by the Biomedical Ethics Board of the Second Xiangya Hospital, China Central South University, and the Biomedical Ethics Board of the Faculty of Health Sciences at the University of Macau (Macao SAR, China).

### Clinical measurements

The current and lifetime histories of psychiatric diagnoses were assessed for all the participants by a professionally trained child psychiatrist using the Schedule for Affective Disorder and Schizophrenia for School-Age Children-Present and Lifetime (K-SADS-PL; Chinese version) [65–67]. The K-SADS-PL is a validated and reliable semi-structured diagnostic interview that is widely used to diagnose psychopathologies in both children and adolescents aged 6 to 18 years in accordance with the DSM-IV criteria [68]. It consists of six sections: 1) an unstructured introductory interview; 2) a diagnostic screening interview; 3) a supplement completion checklist; 4) appropriate diagnostic supplements; 5) a summary of lifetime diagnoses checklist; and 6) the children's global assessment scale [65]. Each individual symptom is scored on a scale of 0-3 rating, where 0 means no information, 1 represents the symptom is not present, 2 implies subthreshold levels of symptomatology, and 3 denotes threshold criteria.

All the CD participants met the K-SADS-PL criteria for CD. By design, none of the CD patients had current or lifetime comorbid psychiatric problems, such as ADHD, anxiety and depression disorders, affective disorders, OCD, oppositional defiant disorder (ODD), mental retardation, alcohol- and drug-use disorder, and substance use disorder. TD healthy controls were screened using the same instrument and were free of all the assessed neurological and psychological disorders. Participants were also excluded if they had any history of neurological disorders such as paralysis, loss of sensation, epilepsy, muscular weakness, seizures, chronic pain, confusion, and prolonged loss of consciousness due to head injury. Finally, all the participants had no contraindications to the MRI environment, no history of head trauma, and did not taken any medicine for at least 3 months prior to participating in the study.

### Impulsiveness assessment

The BIS-11 questionnaire was designed to assess the personality and behavioral traits of impulsiveness [69]. The current version of the BIS-11 consists of 30 items that describe the impulsive or non-impulsive behaviors and preferences, and each item has a four-point scale where 1 stands for rarely or never, 2 denotes occasionally, 3 implies often, and 4 means almost always or always. It consists of three second-order factors of impulsivity: attentional, motor, and non-planning.

### Data acquisition

Data acquisition was performed using a 3T MR scanner (Siemens Allegra, located at the Magnetic Resonance Center of Hunan Provincial People’s Hospital) with an eight-channel head coil. Foam padding was used to minimize the head motion, and earplugs were used to minimize the effect of the scanning noise on brain activation for all subjects. During the resting-state scans, which lasted 5 minutes, all the subjects were instructed to rest quietly with their eyes closed and to relax without thinking of anything or falling asleep. Functional images were obtained using single-shot gradient-recalled Echo Planar Imaging pulse sequences with the following parameters: TR/TE = 3 s/30 ms, flip angle = 90°, field of view = 256 × 256 mm^2^, in-plane matrix = 64 × 64, slice thickness = 3 mm, and no gap. For each subject, a total of 100 volumes (36 slices per volume) of images were collected.

### Data preprocessing

The rsfMRI data were preprocessed using the Data Processing Assistant for Resting-State fMRI (DPARSF) software package [70]. The first three volumes were removed from each participant in favor of the magnetization equilibrium and saturation effects. The remaining 97 consecutive volumes were first slice-timing corrected and then realigned to the first of the remaining volumes for head-motion correction. After these corrections, the motion-corrected functional volumes were spatially normalized into the standard Montreal Neurological Institute (MNI) space with a resampling voxel size of 3 × 3 × 3 mm^3^. Subsequently, to remove the several spurious sources of variance, the CompCor method was used to regress out the six head motion parameters, the averaged signals from white matter signals, and cerebrospinal fluid signals [71]. No global signal was regressed out in order to avoid yielding spurious negative correlations [72]. Finally, the resulting time series were further detrended to remove the linear trend and temporally bandpass filtered in a frequency range of 0.01-0.10 Hz to reduce the effects of low-frequency machine magnetic field drifts and high-frequency physiological noise. No spatial smoothing was applied in order to avoid introducing artificial local spatial correlation according to previous studies [38, 39, 72–76].

Furthermore, to reduce the impact of motion artifacts induced by systematic biases on the time series, a “scrubbing” method and a frame-wise displacement (FD) threshold of 0.5 mm were applied to remove the contaminated time points [77]. Specifically, at each time point *t*, the estimation of motion was computed as FD using the formula $FD_{t}=\left|\Delta fd_{tx}\right|+\left|\Delta fd_{ty}\right|+\left|\Delta fd_{tz}\right|+\left|\Delta fd_{t\alpha }\right|+\left|\Delta fd_{t\beta }\right|+\left|\Delta fd_{t\gamma }\right|$, the three head translations ($d_{tx},d_{ty},d_{tz}$), and the three head rotations ($d_{t\alpha ,}d_{t\beta },d_{\gamma }$). In this equation, $\Delta fd_{tx}=fd_{\left(t-1\right)x}-fd_{tx}$. The calculation is similar for the other rigid body parameters ($d_{ty},d_{tz},d_{t\alpha },d_{t\beta },d_{t\gamma }$).

The angle rotational displacements were converted from degrees to millimeters by applying a radius *r* = 50 mm, which is approximately the mean distance from the center of the MNI space to the cortex. Image time points with *FD_t_* > 0.3 mm were considered potentially contaminated with motion artifacts and excluded from the time series [77]. The root mean squared variance over voxels was also computed to measure how much the intensity of a brain image changes in comparison to the previous time points [77]. Notably, no data exceeded ±2 mm or ±2° for their translational or rotational parameters, and there was no significant group difference in mean FD (*p* = 0.8761) between the CD group (0.107 ± 0.011) and TD group (0.109 ± 0.009) according to two-sample *t*-tests.

### Network construction

A functional brain network consists of nodes and edges between nodes. To determine the nodes and edges of brain functional connectivity networks, the following procedure was applied [78, 79].

### Definitions of nodes and edges

To define the nodes of brain functional connectivity networks, the automated anatomical labeling (AAL) atlas was used to parcellate the whole brain into 90 anatomical regions of interest (ROIs) (45 for each hemisphere, Table 4) [80]. Each ROI represents a node of the network. To determine the edges of brain networks, a 90 × 90 temporal correlation matrix was acquired for each subject by calculating the Pearson correlation coefficients between the time series of all pairs of ROIs. Prior to correlation analysis, the representative time series of each ROI for each subject was obtained by averaging the functional MRI time series of all voxels within that ROI. Finally, Fisher’s r-to-z transformation was applied to the correlation matrices, and individual correlation matrices were thresholded into binarized matrices with a sparsity value (defined as the ratio of total number of edges in a network to the maximum possible number of edges to make sure that networks from two comparison groups had the same number of edges or wiring cost). For an *N* × *N* binary undirected graph *G* (*N* = 90 indicates 90 nodes in the present study), the edges *e_ij_* were defined based on the following graph construction:

$$e_{ij}=\{\begin{array}{ll} 1, & if\left|z_{ij}\right|\geq T\\ 0, & otherwise \end{array},$$

**Table 4:** Anatomical regions of interest (ROIs) and abbreviated regional labels

ROI	Index	ROI	Index
Precentral gyrus	(1,2)	Lingual gyrus	(47,48)
Superior frontal gyrus	(3,4)	Superior occipital gyrus	(49,50)
Superior frontal gyrus, orbital	(5,6)	Middle occipital gyrus	(51,52)
Middle frontal gyrus	(7,8)	Inferior occipital gyrus	(53,54)
Middle frontal gyrus, orbital	(9,10)	Fusiform gyrus	(55,56)
Inferior frontal gyrus, opercular part	(11,12)	Postcentral gyrus	(57,58)
Inferior frontal gyrus, triangular part	(13,14)	Superior parietal gyrus	(59,60)
Inferior frontal gyrus, orbital	(15,16)	Inferior parietal gyrus	(61,62)
Rolandic operculum	(17,18)	Supramarginal gyrus	(63,64)
Supplementary motor area	(19,20)	Angular gyrus	(65,66)
Olfactory cortex	(21,22)	Precuneus	(67,68)
Superior frontal gyrus, medial	(23,24)	Paracentral lobule	(69,70)
Superior frontal gyrus, medial orbital	(25,26)	Caudate nucleus	(71,72)
Gyrus rectus	(27,28)	Putamen	(73,74)
Insula	(29,30)	Pallidum	(75,76)
Anterior cingulate gyri	(31,32)	Thalamus	(77,78)
Median cingulate gyri	(33,34)	Heschl gyrus	(79,80)
Posterior cingulate gyrus	(35,36)	Superior temporal gyrus	(81,82)
Hippocampus	(37,38)	Superior temporal gyrus: temporal pole	(83,84)
Parahippocampal gyrus	(39,40)	Middle temporal gyrus	(85,86)
Amygdala	(41,42)	Middle temporal gyrus: temporal pole	(87,88)
Calcarine fissure	(43,44)	Inferior temporal gyrus	(89,90)
Cuneus	(45,46)		

The odd and even numbers stand for the left hemisphere and right hemisphere brain regions, respectively. The regions are listed according to a prior AAL atlas [80].

If the absolute *z_ij_* (the Fisher *r*-to-*z* of the partial correlation coefficient between node *i* and node *j*) exceeds a predefined threshold *T*, an undirected edge is said to exist; otherwise it does not exist.

### Network analysis

#### Threshold selection

Instead of selecting a single threshold, we applied a range of sparsity values from 0.11 to 0.44 with intervals of 0.01 according to criteria suggested in previous studies [78]. Specifically, 1) the minimum sparsity was selected to assure that the mean degree across all nodes of each thresholded network was larger than 2 × log(*N*), where *N* = 90 is the number of nodes; 2) the maximum sparsity was selected to ensure that the small-worldness scalar of each thresholded network was larger than 1.1 for all the participants.

### Small-world network measurements

The graph theoretical analysis was carried out using the graph theoretical network analysis (GRETNA) toolbox (Version 1.2, https://www.nitrc.org/projects/gretna/) to calculate the topological properties and evaluate the functional connectivity organization [81, 82]. The clustering coefficient of a node *i* is defined as the ratio of the number of existing connections among the node’s neighbors to the number of all possible connections in the subgraph *G*_*i*_ [83]:

$$C_{i}=\frac{E_{i}}{K_{i}\left(K_{i}-1\right)/2}\;$$

in which *E*_*i*_ and *G*_*i*_ denote the number of edges and nodes in subgraph *G*_*i*_, respectively. The clustering coefficient of a functional connectivity network is the average of the clustering coefficients of all nodes:

$$C_{p}=\frac{1}{N}\sum_{i\in G}C_{i},$$

which measures the local interconnectivity of a network.

The mean shortest path length of a node *i* is defined as:

$$L_{i}=\frac{1}{N-1}\sum_{i\neq j\notin G}\min\left|L_{ij}\right|,$$

where min $\left|L_{ij}\right|$ is the absolute shortest path length (i.e., the smallest number of edges traversed between two nodes) between node *i* and node *j*. The mean shortest path length of a network is then the average of the shortest path lengths between the nodes:

$$L_{p}=\frac{1}{N}\sum_{i\in G}L_{i}$$

The normalized clustering coefficient $\gamma =\frac{c_{p}}{c_{random}}$ and normalized characteristic path length $\lambda =\frac{L_{p}}{L_{random}}$ were computed, where cp and Lp indicate the mean clustering coefficient and shortest path length of the functional connectivity network, respectively. $C_{random}$ and $L_{random}$ represent the mean clustering coefficient and shortest path length of 100 matched random networks that preserve the same numbers of nodes, edges, and degree distribution as the real network [84, 85]. The small-world network properties (clustering coefficient *C*_*p*_ and path length *L*_*p*_) were first proposed by Watts and Strogatz [17]. To quantify the small-world characteristics, 100 random networks were generated at each sparsity threshold for each individual network with the same degree distribution as that of the functional connectivity networks using a Markov-chain algorithm [86]. Typically, we scaled *C*_*p*_ and *L*_*p*_ of the examined functional connectivity networks with the averaged $C_{random}$ and $L_{random}$ of all 100 random networks (i.e., normalized clustering coefficient lambda γ=CpCrandom and normalized characteristic path length gamma λ=LpLrandom). A real network can be considered as a small-world network if it fulfills the following criteria: *γ* > 1 and *λ* ≈ 1 [17], or the small-wordness scalar σ=λγ>1.1. In other words, the small-world network has a higher clustering coefficient and a similar path length compared to a random network [87, 88].

### Regional nodal measurements

The regional nodal properties can be characterized by a number of key measurements, including the nodal degree *Deg*_*i*_, nodal efficiency *E*_*nodal*_, and the nodal betweenness *BC*_*i*_, [38, 89]. The nodal degree is defined as the number of nodes in subgraph *G*_*i*_, which is the graph including the nodes that are direct neighbors of node *i*. The nodal degree evaluates the extent to which the node is connected to the rest of the other nodes in a network. The nodal efficiency *E*_*nodal*_ is the inverse of the harmonic mean of the length between node *i* and all other nodes in the network. It is used to deal with disconnected graphs or non-sparse graphs and measures the level of information propagation of a node with all other nodes in the network. The nodal betweenness is defined as the fraction of all shortest paths in the network that pass through node *i*. It estimates the influence of a node over information flow with the rest of the nodes in a network.

### Efficiency of small-world networks

The network efficiency can be described in terms of global efficiency *E*_*glo*_ and local efficiency *E*_*loc*_. The global efficiency *E*_*glo*_ of a network is the inverse of the harmonic mean of the shortest path length between each pair of nodes [38, 89]:

$$E_{glo}=\frac{1}{N\left(N-1\right)}\sum_{i\neq j\in G}\frac{1}{\min\left|L_{ij}\right|}$$

where min $\left|L_{ij}\right|$is the absolute shortest path length between node *i* and node *j* in network *G*. It indicates the capability of parallel information transfer through the whole network.

The nodal efficiency *E*_*nodal*_ of a node *i* is calculated as:

$$E_{nodal}\left(\text{i}\right)=\frac{1}{N-1}\sum_{j,K\in G}\frac{1}{\min\left|L_{jk}\right|}$$

The local efficiency *E*_*loc*_ denotes the mean of all the local efficiencies of the nodes in subgraph *G*_*i*_:

$$E_{loc}=\frac{1}{N}\sum_{i\in G}E_{nodal}\left(i\right),$$

where *E*_*nodal*_*(i) = E*_*glo*_*(G*_*i*_*)*. Since node *i* is not an element of subgraph *G*_*i*_, the local efficiency can also be considered as a measure of the fault tolerance of the network, suggesting how well each subgraph exchanges information when node *i* is eliminated [38]. For each network, we computed six global network properties ($C_{p},L_{p},\lambda ,\gamma ,E_{glo}$ and *E*_*loc*_) and three regional nodal measurements (*Deg*_*i*_, *E*_*nodal*_ and *BC*_*i*_). For each network index, we further obtained a summarized scalar for topological characterization of brain functional networks by computing the AUC [78]. The integrated AUC of a network metric Y was computed over the sparsity threshold range of *S*_*1*_ to *S*_*n*_ with intervals of Δ*S*:

$$Y^{AUC}=\sum_{k=1}^{n-1}\left[Y\left(S_{k}\right)+Y\left(S_{k+1}\right)\right]\times \Delta S/2$$

We also computed the cost efficiency, which is defined as the difference between the global efficiency and the cost (sparsity); i.e., *E*_*glo*_ - cost, which would be positive in the case of an economical network [38].

### Statistical analysis

#### Differences in the network properties

To compare the global network topological properties (*C*_*p*_, *L*_*p*_, *E*_*glo*_, *E*_*loc*_, λ, γ, and σ) between the CD and TD groups, a series of two-sample *t*-tests (*p* < 0.05, uncorrected) were performed for each property across the preselected sparsity threshold range of 0.11 ≤ *T* ≤ 0.44 with intervals of 0.01. To evaluate the alterations of the regional nodal properties (*Deg*_*i*_, *E*_*nodal*_ and *BC*_*i*_) between the two groups, nonparametric permutation tests were also performed on the AUC of each nodal property (5000 iterations, *p* < 0.05, uncorrected, controlling for the age, and mean FD) [90]. Briefly, we first computed the actual between-group differences of the AUC of each network metric. We then we put this difference into a null permutation distribution of differences to recalculate by chance through randomly assigning the values of each subject to two randomized groups with the same size as the CD and TD groups. This procedure was repeated for 5,000 permutations. A *p*-value < 0.05 for multiple comparisons was considered to indicate a significant difference for all AUCs of each network topology characteristic.

### Correlations between the network properties with BIS-11 scores across all the subjects

Pearson’s correlation was used to assess the relationship between the global network properties and the BIS-11 scores (including the total and three subtype scores) in the CD and TD groups with age and mean FD as confounding factors (independent variables: AUC of each network property; dependent variables: BIS-11 scores of both the CD and TD groups). We also applied Pearson’s correlation to investigate the relationship between the AUCs of nodal network properties in brain regions that showed significant differences in any nodal network properties and BIS-11 scores across all subjects.The significant threshold applied for false-positive correction for all of the analyses was calculated as one divided by the number of nodes, which resulted in 1/90 or 0.011.

### Relationships between the nodal network properties with BIS-11 scores in CD group

Pearson’s correlation was also used to assess the relationship between the nodal network properties and the BIS-11 scores (including the total and three subtype scores) in the CD group with age and mean FD as confounding factors (independent variables: AUC of each network property; dependent variables: BIS-11 scores of the CD group). A value of 0.011 was applied as a significant threshold for false-positive correction for all of the analyses.

## CONCLUSIONS

In summary, this is the first study to investigate the topology organization of brain functional networks in patients with CD using rsfMRI and graph theoretical approaches. Our results suggested that the CD patients as well as the healthy controls showed small-world properties, thus providing further evidence for the presence of small-world characteristics in complex brain functional networks. Moreover, altered global and local efficiency and the altered nodal topology were found in CD patients, suggesting that the disorder has a serious impact on the topological properties of brain functional connectivity networks. Importantly, correlations between the BIS-11 scores and the aberrant topology organization in the fronto-limbic-striatum systems were found in this study, which shed light on the abnormalities of the fronto-limbic-striatum systems in CD patients. Taken together, our results provide novel insights into understanding the pathophysiology underlying CD.
